# Validity and Reliability of the Turkish Chronic Pain Acceptance Questionnaire

**DOI:** 10.4274/balkanmedj.2016.1998

**Published:** 2018-05-29

**Authors:** Hazel Ekin Akmaz, Meltem Uyar, Yasemin Kuzeyli Yıldırım, Esra Akın Korhan

**Affiliations:** 1 Clinic of Anesthesiology and Reanimation, Manisa State Hospital, Manisa, Turkey; 2 Department of Algology, Ege University School of Medicine, İzmir, Turkey; 3 Ege University School of Nursing, İzmir, Turkey; 4 Department of Nursing, İzmir Katip Çelebi University School of Health Science, İzmir, Turkey

**Keywords:** Acceptance, chronic pain, methodological study, reliability, Turkish, validity

## Abstract

**Background:**

Pain acceptance is the process of giving up the struggle with pain and learning to live a worthwhile life despite it. In assessing patients with chronic pain in Turkey, making a diagnosis and tracking the effectiveness of treatment is done with scales that have been translated into Turkish. However, there is as yet no valid and reliable scale in Turkish to assess the acceptance of pain.

**Aims:**

To validate a Turkish version of the Chronic Pain Acceptance Questionnaire developed by McCracken and colleagues.

**Study Design:**

Methodological and cross sectional study.

**Methods:**

A simple randomized sampling method was used in selecting the study sample. The sample was composed of 201 patients, more than 10 times the number of items examined for validity and reliability in the study, which totaled 20. A patient identification form, the Chronic Pain Acceptance Questionnaire, and the Brief Pain Inventory were used to collect data. Data were collected by face-to-face interviews. In the validity testing, the content validity index was used to evaluate linguistic equivalence, content validity, construct validity, and expert views. In reliability testing of the scale, Cronbach’s α coefficient was calculated, and item analysis and split-test reliability methods were used. Principal component analysis and varimax rotation were used in factor analysis and to examine factor structure for construct concept validity.

**Results:**

The item analysis established that the scale, all items, and item-total correlations were satisfactory. The mean total score of the scale was 21.78. The internal consistency coefficient was 0.94, and the correlation between the two halves of the scale was 0.89.

**Conclusion:**

The Chronic Pain Acceptance Questionnaire, which is intended to be used in Turkey upon confirmation of its validity and reliability, is an evaluation instrument with sufficient validity and reliability, and it can be reliably used to examine patients’ acceptance of chronic pain.

Chronic pain is defined as pain that lasts for more than 3 months and continues beyond the expected recovery process. Today, it is a universal problem with a significant effect on affected individuals’ psychosocial state, quality of life, and functional abilities. The annual cost of  pain management is as high as 60 billion dollars a year, which is more than the yearly costs for cardiovascular diseases, and also it causes the loss of an estimated 700 million working days (1,2,3,4). Chronic pain is an illness that affects behavior and lifestyle (5,6). Effective evaluation of the pain level a patient is experiencing has critical importance for determining planned intervention methods related to treatment of the pain. It is necessary to use a common language to measure the experience of pain, which has a negative effect on the patient’s life, disrupts quality of life, and is associated with a large number of pathologies (7,8,9,10,11,12). Today, many scales are used in the assessment of pain. In Turkey, doctors and nurses assessing pain generally use scales developed in other countries that have been adapted to the Turkish language. For example, McGill Pain Questionnaire short form and visual analog scale is often used by nurses for evaluation for pain management. In international publications, the translation into Turkish and use of a scale that is well known and about which there is a stock of data saves time that a healthcare worker would otherwise spend in preparing a new scale, and provides ease of communication and comparable data (13).

In Turkey, in assessing patients with chronic pain, making a diagnosis, and tracking the effectiveness of treatment, use is made of scales that have been translated into Turkish (8,9,14,15). Recently, studies have been focused on pain control with the concepts of accepting pain, being able to live with pain, and voluntarily accepting pain rather than trying to control pain (16,17,18,19,20,21,22). However, there is as yet no valid and reliable scale in the Turkish language for assessment of the acceptance of pain. This scale [Chronic Pain Acceptance Questionnaire (CPAQ)] also reveals the patient’s emotional changes related to pain as well as the related effects on physical function. In this sense, the scale will make the multidimensional evaluation of the chronic pain possible. Also, owing to functional assessment, the validity and reliability study was conducted in many languages. Bernini et al. (23) studied the validity and reliability of the scale in Italian, Rodero et al. (24) in Spanish, Cho et al. (25) in Korean, Mesgarian et al. (26) in Persian, Liu et al. (27) in Chinese, and Hilde et al. (28) in Norwegian. In the light of this information, the objective of the present study was to test the validity and reliability of the Turkish version of the  CPAQ, which has previously been translated into many languages.

## MATERIALS AND METHODS

The objective of this methodological and cross-sectional study was to establish the validity and reliability of the Turkish version of the CPAQ. A patient identification form, the CPAQ, and the Brief Pain Inventory were used to collect data. The patient identification form was used to gather information on age, sex, education level, profession, location of pain, duration of pain, diagnosis, and treatment received. The CPAQ was developed by MacCallum et al. (29). The Cronbach’s α coefficient of the scale is 0.78. The scale is a 7-point Likert-type scale with 20 items and 2 subscales. The first subscale, activity engagement, consists of 11 items and evaluates how much the presence of pain affects the patient’s daily activities. The second subscale, pain willingness, is composed of nine items and evaluates the extent to which pain can be tolerated without an attempt to avoid or control it.

In the first stage of our research, studies were conducted first on the language, structure, and content validity in the validity and reliability studies of the CPAQ. To ensure language validity, the necessary permission was first obtained from the researchers who developed the scale, and then the scale was translated from English into Turkish, first by the researcher, then by a translator who knew both languages (Turkish and English), and finally by 10 teachers, experts, and researchers employed in the anesthesiology and reanimation department of a university hospital. The final version, formed by selecting the most suitable expressions from among the Turkish translations, was then translated back into English by another translator who understood and was a fluent speaker of both languages. The items on the retranslated scale was compared with the original, and after necessary revisions by the researcher and the thesis advisor, the final version of the scale was presented for expert review for testing of language and culture equivalency as well as content validity, and content validity was assessed by four anesthetists and two algologists (Table 1).

In this study, the construct validity of the scale was evaluated by means of explanatory factor analysis. In the reliability testing of the scale, the test-retest technique was used to determine the criterion of temporal invariability, and the scale was applied again with 30 patients 2 weeks after the first measurement (Table 2).

The research was conducted between November 2013 and February 2014 in the algology department outpatient clinic of a university hospital. The research sample consisted of patients attending the algology department between November 2013 and February 2014 who agreed to take part in the research, were over the age of 18, were educated to at least primary school level, had had chronic pain for at least 3 months, could communicate in and speak Turkish, did not have a hearing problem, and did not have any cognitive disorder. In determining the sample size for the study, account was taken of the number of items (n=19) in the CPAQ. In the literature, 7-10 people are recommended for each item to determine the size of the sample, and therefore a sample group of 140-200 individuals were planned when the study was started (29). The study ultimately was completed with 201 patients. For test-retest reliability, the CPAQ was repeated with 30 patients 2 weeks after the first application.

Data gathered in the research were analyzed SPSS version 16.0 software (SPSS, Chicago, IL, USA). In the analysis of data, numbers, percentages, the Pearson product-moment correlation coefficient, the Cronbach’s α reliability coefficient, Guttman split-half reliability coefficient, and the Spearman-Brown prediction formula were calculated.

Written permission was obtained to conduct the research from the ethics committee of the university hospital where the research was conducted, as well as from the anesthesiology and reanimation department and the algology department of the hospital where the research was conducted. The purpose of the study, the methods, and the expected results were explained to the participants, and their written permission was obtained.

## RESULTS

The mean age of the included patients was 54.69±14.40 years, and 69.7% were female. In terms of diagnosis, 41.8% had lumbar hernia and 15.9% had degenerative joint disease. The mean duration of pain in the patients was 70.33±104.33 months, and 43.8% of the patients were using nonsteroidal anti-inflammatory medicines (Table 3).

Kendall’s coefficient of concordance (W) for the expert views taken for the CPAQ was calculated to be 0.593. This coefficient was greater than 0.05, showing that the views of the six experts were in concordance. Analysis of the basic components of the factor structure of the CPAQ (i.e., the subscales) was done using varimax rotation, and it was established that, according to the results of explanatory factor analysis of the construct validity of the scale, the scale items could be grouped under two factors that explained 56.33% of the total variance. The variance loads explained by the factors were calculated as 28.98 for the first factor and 27.35 for the second factor. The factor loads for the scale items varied between 0.41 and 0.79 (Table 4). The Kaiser-Meyer-Olkin (KMO) test of sampling adequacy value was 0.880, with a statistically significant Bartlett sphericity (p=0.01).

The correlation coefficient (r) obtained as a result of the reapplication of the scale to 30 patients after a space of 2 weeks was 0.887 and was statistically significant (p=0.000). The test-retest correlation coefficient of the activity engagement subscale was 0.890 and that of the pain willingness subscale was 0.841, both of which were statistically significant (p=0.000). These results showed that the consistency of the scale over time was adequate. It was determined that, in terms of the internal consistency analysis of the CPAQ and its subscales, split-half reliability analysis, item-total correlation analysis, and Cronbach’s α coefficient analysis, the correlation coefficients were between 0.888 and 0.904. The reliability coefficient of one-half of the scale 0.816. The reliability coefficient of the whole scale was calculated using the Spearman-Brown formula, and this value was determined to be 0.899.

The Cronbach’s α reliability coefficient of the CPAQ was calculated to be 0.94. The Cronbach’s α coefficient of the activity engagement subscale of the CPAQ was found to be 0.911 and that of the pain willingness subscale was 0.89. Item-total correlation scores of the CPAQ were determined to be between 0.47 and 0.79 (Table 5). The correlation between CPAQ scores and the scores on items 3, 4, 5, 6, 8, and 9 of the Schedule Performance index (SPI) was calculated using Spearman’s correlation coefficient. The correlation coefficient between scores for eight items of the SPI and CPAQ was found to be 0.75, but this correlation was not statistically significant (p>0.05). The correlation coefficients between the CPAQ and the other items of the SPI were between -0.566 and -0.244 and were statistically significant (p<0.05). The correlation coefficient of the activity engagement subscale and item 8 of the SPI was calculated to be 0.60, but it was not statistically significant (p>0.05). The correlation coefficients of this subscale and the other SPI items were between -0.533 and -0.207 and were statistically significant (p<0.05). The correlation coefficient between the pain willingness subscale and item 8 of the SPI was 0.84, but it was not statistically significant (p>0.05). The correlation coefficients of this subscale and the other SPI items were between -0.539 and -0.258 and were statistically significant (p<0.05).

The mean scores of the CPAQ items varied between 1.09 and 4.01, and the standard deviations varied between 1.23 and 1.85. The mean score of the activity engagement subscale was 30.70 and that of the pain willingness subscale was 13.60. The mean CPAQ score was 21.78; the lowest score was 8.00, and the highest was 106.00.

## DISCUSSION

A growing body of empirical evidence suggests that acceptance plays an important role in coping with chronic pain. Pain acceptance is the process of giving up the struggle with pain, remaining active, and learning to live a meaningful life despite pain. This study shows that the Turkish translation of the CPAQ has satisfactory psychometric properties in our sample of patients with long-lasting chronic pain.

When developing a scale or adapting it to Turkish, reliability and validity work is performed by basic psychometric testing (30). It would be wrong to use an instrument that could not make a correct or reliable assessment or that could make a correct assessment but did not serve the purpose for which it was used. This makes it necessary to consider both the reliability and the validity of a measurement instrument. Even though the validity of an assessment instrument is dependent on its reliability, an instrument that is reliable but not valid is of little use in practice (31). Content validity is the degree to which the items on a measurement instrument represents, in a balanced way, the topics that the instrument aims to assess. For this reason, the characteristic to be assessed must be well sampled. For this purpose, the basic task is to define what is included in the instrument. Expert views are taken on whether the questions on the measurement instrument are suitable for its purpose and whether they represent the field that is to be assessed (32,33).

In this study, language equivalence, content validity, and construct validity were used to test the validity of the scale. In the assessment of the experts’ evaluation scores by Kendall’s W analysis, we observed that there was no statistical difference between the experts’ scores and that there was concordance between the experts. In light of the experts’ views, it can be said that the revised and corrected scale is suitable for its purpose and that it represents the field that is to be measured. In this way, a measure of content validity has been provided as a result of the expert views and recommendations. To determine whether any further corrections were needed for the scale items, the scale was piloted with 10 patients. The data derived from these 10 patients was not included in the study (34).

Reliability is the basic characteristic that any measurement instrument must have. It is the ability of a measurement instrument to measure without any errors. This characteristic determines whether the instrument collects data correctly and whether it can be repeated. For reliability analysis, the correlation coefficient of the items in the measurement instrument is calculated in order to determine to what extent the scale items are related to the scale as a whole and that are frequently used in the choice of item. In item analysis, the correlation is calculated between the total score obtained by respondents and the total score on each item. If the correlation of an item with the total score is low, that item may be judged as measuring a different characteristic from the other items. A low item-total correlation has the effect of lowering reliability, and therefore such items are removed from the scale. In our study, it was found as a result of the item analysis performed to determine the internal consistency of the scale that the item-total correlations of all the items on the scale were at an adequate level (32,34,35).

Because the evaluation measure of the scale is itself, it is very important that the scale should be consistent within itself. The α coefficients of a scale composed of items that show a high correlation with each other are high. The Cronbach’s α coefficient is a measure of the homogeneity and internal consistency of the items on the scale. A high Cronbach’s α coefficient means that the items on the scale are consistent with each other on that scale and that the scale is composed of items that focus on the same factor. In establishing the level of reliability of a scale, it is recommended that in conditions where the item scores in the item solution are continuous (Likert-type), the Cronbach’s α coefficient should be calculated (34). In this study, the Cronbach’s α coefficient was evaluated to calculate the internal consistency coefficient of the CPAQ, which is a Likert-type scale. The internal consistency reliability coefficient of the 20-item CPAQ was found to be 0.94, and the scale had a high level of reliability. The internal consistency reliability coefficients of the subscales varied between 0.89 and 0.91. It was concluded that the values that were obtained were at an acceptable level and that the items on the scale were consistent with each other and were formed from items focused on the same factor. In other words, the level of homogeneity of the scale was adequate. In the original validity and reliability testing of the scale, Cronbach’s α was 0.91 and that of the subscales varied between 0.83 and 0.89. The scale and subscales showed a higher level of internal consistency than the original form of the scale. It is thought that this may arise from the nature of the pain experienced by patients and from intercultural differences.

The reliability coefficient obtained by the method of dividing the scale into two halves is known as equivalent split-half reliability. In calculating this coefficient, if the scale is one-dimensional, it is performed for the scale as a whole, whereas if there are subscales, each subscale is taken as a whole, and the analysis is carried out within the subscales. It is the most used of the methods to establish scale reliability. To obtain the reliability coefficient of the test as a whole, an equation developed by Spearman and Brown was used (33). In the split-half reliability analyses of the CPAQ, the Cronbach’s α coefficient, the Spearman-Brown coefficient, and the Guttman split-half coefficient were found to be at high levels. These results show that the scale has acceptable internal consistency and that it is a reliable scale.

Explanatory factor analysis was performed for construct concept validity. Factor analysis is an operation dependent on the correlation of items with each other, and it is performed to assess whether the items on the scale gather under different dimensions. The objective in factor analysis is to express the Turkish validity and reliability of the multiple CPAQ items in less than 20 items. In explanatory factor analysis, there are various methods of determining the factor numbers. The most often used of these is the technique of taking the factors with eigenvalues greater than 1, known as the Kaiser-Guttman rule. In determining which item belongs to which factor, the measure is the factor load showing the degree of correlation between a factor and an item. When applying factor analysis, the consistency and the adequacy of the sample must be taken into account. When performing factor analysis, the adequacy of the sample is decided by looking at the KMO value. For a good factor analysis, a KMO value greater than 0.60 is required (34). In the present study, the analysis of sample adequacy calculated as the KMO value was found to be quite adequate for factor analysis. The findings obtained showed that the sample size used was adequate and that the data were suitable to perform factor analysis.

This study provides support for the reliability and validity of the Turkish version of the CPAQ. Our findings were consistent with those obtained in prior studies and other versions of the CPAQ (23,24,25,26,27,28). However, Nicholas and Asghari (36) found that the incremental validity of the CPAQ was insufficient after controlling for other variables, which were a combination of pain self-efficacy, pain-related anxiety, and catastrophizing.

The CPAQ is an instrument that enables assessment of pain acceptance in persons with chronic pain. This scale also reveals the patient’s perceptions related to life changes and provides an evaluation of the emotional changes and impairment of physical function that patients experience because of the pain. The multidimensional evaluation of pain enables health professionals to provide pain management more effectively. Clinicians, as a result of their evaluations using the scale, can make better decisions for interventions required for their patients, and they can develop new approaches according to patients’ changing situations. Also, the CPAQ may be a valuable clinical tool reflecting changes in pain acceptance during treatment, which in turn is likely to be a useful strategy for treatment development.

In conclusion, the CPAQ, which is intended to be brought to Turkey on the basis of validity and reliability analyses, is an evaluation instrument with sufficient validity and reliability to be reliably used for evaluating pain acceptance levels of patients who experience chronic pain. Use of this scale can reveal how patients’ pain affects their lives, which will play a key role in managing patients with chronic pain. It is suggested that the scale also can be used for different chronic pain syndromes in accordance with all of these implications.

## Figures and Tables

**Table 1:**
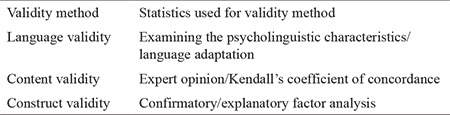
Validity methods

**Table 2:**
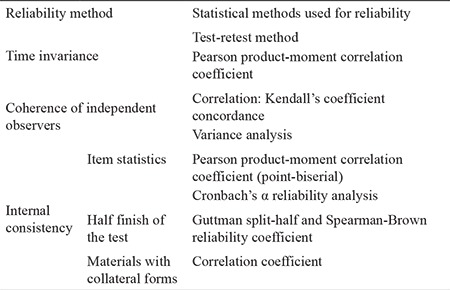
Reliability methods

**Table 3:**
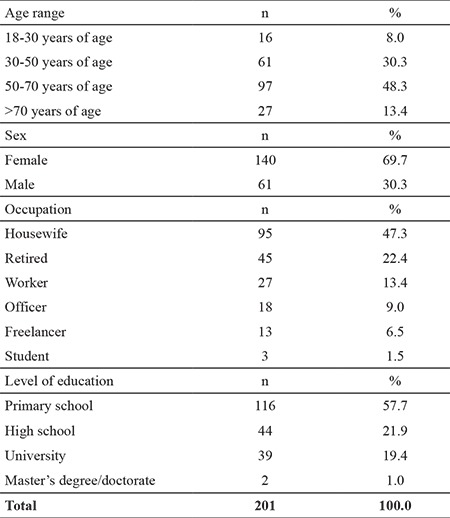
Distribution of the patients according to their descriptive characteristics

**Table 4:**
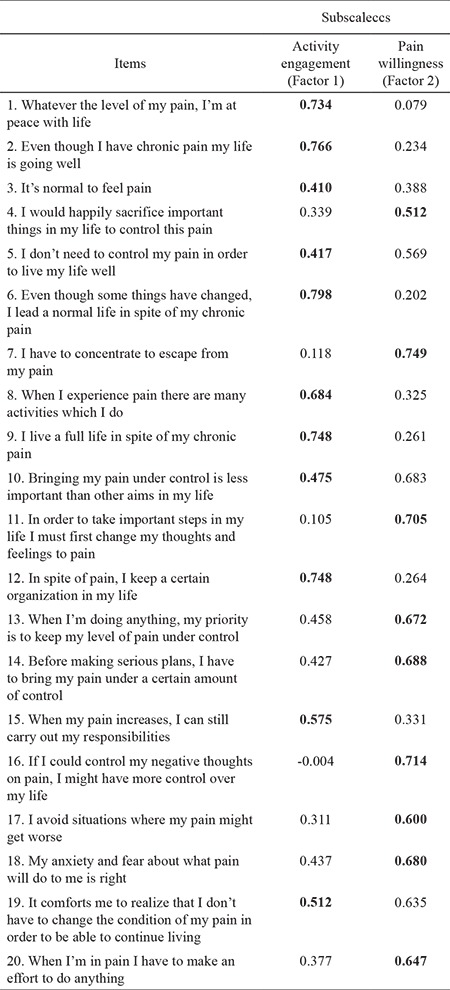
Explanatory factor analysis of Chronic Pain Acceptance Questionnaire

**Table 5:**
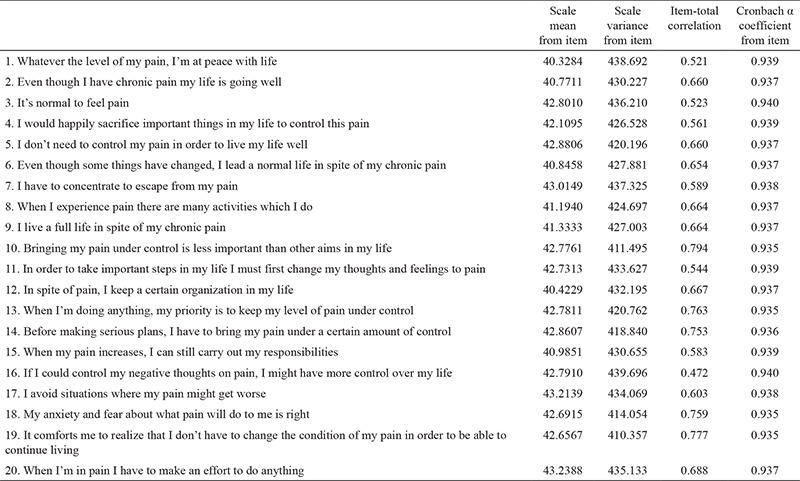
Analysis results of Chronic Pain Acceptance Questionnaire items

**Chronic Pain Acceptance Questionnaire:**
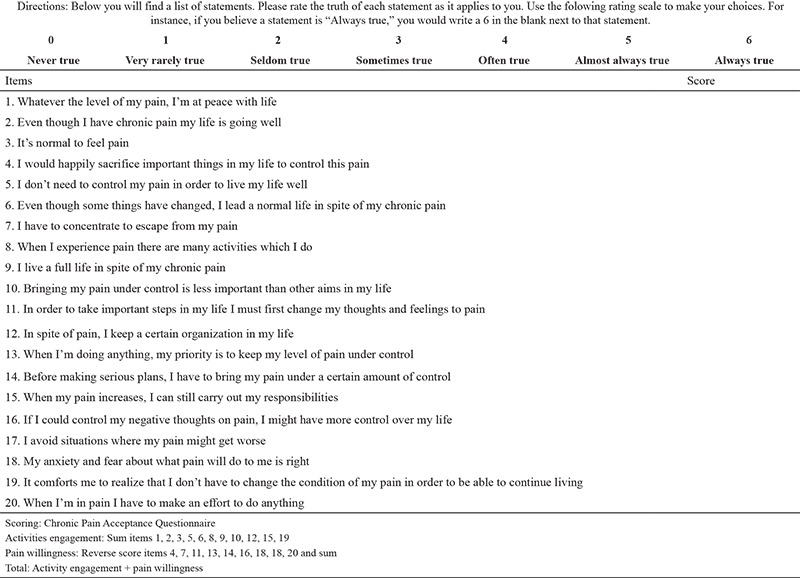
Directions: Below you will find a list of statements. Please rate the truth of each statement as it applies to you. Use the folowing rating scale to make your choices. For instance, if you believe a statement is “Always true,” you would write a 6 in the blank next to that statement.
